# Adult Cranberry Beverage Consumers Have Healthier Macronutrient Intakes and Measures of Body Composition Compared to Non-Consumers: National Health and Nutrition Examination Survey (NHANES) 2005–2008

**DOI:** 10.3390/nu5124938

**Published:** 2013-12-04

**Authors:** Kiyah J. Duffey, Lisa A. Sutherland

**Affiliations:** 1 Department of Human Nutrition, Foods and Exercise, Virginia Tech, 223 Wallace Hall, 295 Campus Drive, Blacksburg, VA 24061, USA; 2 LA Sutherland Consulting, LLC, 6 Mink Drive, Hanover, NH 03755, USA; E-Mail: la@lasutherlandgroup.com

**Keywords:** adults, obesity, cranberry, sugar sweetened beverages, flavonoids

## Abstract

Flavonoids, present in high levels in cranberries, are potent bioactives known for their health-promoting benefits, but cranberry beverages (CB) are not typically recommended as part of a healthy diet. We examine the association between CB consumption with macronutrient intake and weight status. Data for US adults (≥19 years, *n* = 10,891) were taken from the National Health and Nutrition Examination Survey (NHANES) Survey 2005–2008. Total CB consumption was measured over two non-consecutive 24-h dietary recalls. Linear and logistic regression models adjusting for important covariates were used to examine predicted differences between CB consumers and non-consumers on macronutrient and anthropometric outcomes. Results are weighted to be nationally representative. CB consumers (*n* = 581) were older (>50 year) non-Hispanic black females. They consumed an average 221 mL (7.5 oz) CB per day. In fully adjusted models CB consumers (*vs.* non-consumers) had higher carbohydrates and total sugars and lower percent energy from protein and total fat (all *p* < 0.001), but no difference in total energy. A significantly higher proportion of CB consumers were predicted to be normal weight (BMI < 25 kg/m^2^; *p* = 0.001) and had to have lower waist circumferences (*p* = 0.001). Although there was not a significant trend across level of CB intake, low and middle level CB consumers compared to non-consumers were more likely to be normal weight (*p* < 0.001) and less likely to be overweight/obese (BMI ≥ 25 kg/m^2^, *p* < 0.001). Despite having slightly higher daily macronutrient intakes, CB consumers have more desirable anthropometric measures compared to non-consumers.

## 1. Introduction

Total energy [1,2,3] and total and added sugar [4,5] intake have been steadily increasing over the last 30 years, although levels appear to have leveled or be decreasing among certain segments of the population. Consistently, sugar-sweetened beverages (SSB) (namely carbonated soft drinks, or sodas) account for the largest proportion of added sugar consumption among adults [4,6,7]. Although some observational and epidemiological studies have found negative associations with health outcomes including weight gain and obesity, diabetes, and the metabolic syndrome [8,9,10,11,12,13,14], a recent meta-analysis of randomized controlled trials (RCT) reported that the studies supporting the link between SSB and obesity are not definitive and more RCT studies are needed [15].

Although fruit drinks are often lumped into the SSBs category for the purpose of observational or intervention studies, 100% fruit juice is typically treated differently as the sugars are all endogenous and juices contain micronutrients and vitamins vital to human health. Orange juice (and to a lesser extent apple juice) is the most commonly studied 100% juice in the published literature, and the most commonly consumed juice [16,17] in the United States. Importantly, 100% fruit juice does not necessarily have the same negative association with health for children or adults in either cross sectional [16] or longitudinal [18,19] studies.

Unlike most other fruits commonly consumed in juice form, cranberries are extremely tart in nature due to low sugar and high acid content. Additionally, flavonoids impart astringency to the flavor, thus requiring the addition of sweeteners for palatability resulting in a range of juice content, averaging around 27%. This makes cranberries particularly susceptible to dual criticism from an added sugar and SSB perspectives. However, cranberry products contain crucial flavonoids important to health at levels similar or higher than many commonly consumed 100% juices. Although numerous clinical studies evaluating the association between cranberry juice consumption with urinary health [20,21,22,23,24,25,26], antioxidant status [27,28], glycemic response [29,30], or cardiovascular health have been conducted [31,32,33] studies evaluating trends in the consumption of, or association with, other markers of health (e.g., anthropometry, dietary status) do not exist. Determining who cranberry juice consumers are and the extent to which their dietary patterns and health outcomes are similar or dissimilar to non-consumers is crucial for understanding the role that cranberry juice plays in promoting human nutrition and in the development of a healthy lifestyle.

## 2. Experimental Section

### 2.1. Study Population

Nationally representative data from the National Health and Nutrition Examination Survey (NHANES) 2005–2008 for adults aged ≥19 years old were used (*n* = 10,891). Individuals with missing data on cranberry juice consumption (*n* = 557) were excluded from all analyses. Sampling methods for continuous NHANES datasets are described in detail elsewhere [34].

### 2.2. Dietary and Macronutrient Intake

Dietary data were collected via two non-consecutive interviewer administered 24-h recalls. The first is conducted in person by trained interviewer during the in person medical exam in the Mobile Exam Center (MEC). All participants were asked to complete an additional 24-h recall over the telephone 3–10 days following the MEC exam. Detailed explanations of the dietary intake data collection are outlined elsewhere [35,36]. Both dietary recalls were conducted using the United State Department of Agriculture’s Automated Multiple Pass Method. Data for both days of intake were used in the present analyses.

Absolute measures of total energy (kcal/day), protein, carbohydrate, total fat, and total sugar (all g/day) were calculated for all participants. Relative measures of intake, percent of daily energy from protein, carbohydrates and total fat, were also calculated.

Cranberry beverage consumption was defined as the total 2-day intake for 100% cranberry juice, cranberry juice cocktail, low-calorie cranberry juice cocktail, and blended cranberry drinks. The daily average intake was also calculated. The cut points <8 oz/day, 8–16 oz/day, and ≥16 oz/day were used to identify individuals as low, middle, and high consumers.

### 2.3. Anthropometrics

Body measurement data were collected by trained health technicians during the physical exam component of the NHANES survey using standard protocols and procedures [37]. Body weight was measured in kilograms using a digital weight scale with the participant wearing light clothing. Height was measured using a standing stadiometer. Waist circumference was measured just above the iliac crest to the nearest 0.1 cm.

### 2.4. Covariates

Data on the covariates of interest were self-reported and collected during in-person interviews by trained technicians. Sociodemographic and lifestyle covariates of interest for these analyses include age, gender, ethnicity (Non-Hispanic White, Non-Hispanic Black, Hispanic, Mexican American), highest level of education completed (<High School, High School/General Equivalency Diploma (GED), Some College, ≥College Degree), and total physical activity (daily metabolic equivalents).

### 2.5. Statistical Analysis

All analyses were conducted using Stata [38]. Differences in sociodemographic and lifestyle characteristics (percent (standard error (SE))) between CB consumers and non-consumers were compared using chi-squared tests, with *p* < 0.05 set for statistical significance. Multivariate adjusted estimates of macronutrient and anthropometric outcomes were determined using linear (macronutrient and anthropometrics) and logistic (BMI distribution) regression models adjusting for age, gender, race/ethnicity, highest level of education completed, total physical activity, and total energy intake (excluding models where macronutrient intake is expressed as a percent of total energy). From these multivariable adjusted model coefficients, values for levels of each outcome for CB consumers and non-consumers were obtained using the PREDICT command in Stata. Differences in these fully adjusted, predicted outcome values were compared between consumers and non-consumers using student’s *t*-tests (continuous outcomes) and chi-squared tests (BMI distribution). Roughly 42% of the sample was missing information on education (*n* = 7640), and 35.9% missing data on physical activity (*n* = 6498). Rather than exclude those individuals we created a variable to account for having missing information on these two variables. Individuals (*n* = 1029) were also missing information on total energy intake. Because total energy may be differentially associated with our exposure (CB consumption) and the outcomes of interest, we excluded these individuals from all models using listwise deletion. All results are weighted to be nationally representative.

## 3. Results

Cranberry beverage consumption by the 581 CB consumers (5.6% of the total sample) averaged 211 ± 8.1 mL/day or roughly 7.5 ± 0.3 fl oz/day (Table 1). Compared to non-consumers, CB consumers were more likely to be Non-Hispanic Black (*p* < 0.001) woman (*p* = 0.002) over the age of 50 (*p* = 0.032) in self-reported “very good” (*p* = 0.008) or “fair” (*p* = 0.010) health. CB consumers were also less likely than non-consumers to smoke “every day” (*p* = 0.013, Table 1).

**Table 1:** Sociodemographic and lifestyle characteristics among adult cranberry beverage consumers * and non-consumers, National Health and Nutrition Examination Survey (NHANES) 2005–2008.

Demographics	Cranberry Beverage		
	Non-Consumers *n* = 9753	Consumers *n* = 581	*P*-Value ^†^
	% (SE)	% (SE)	
**Gender**			
Men	49.0 (0.4)	42.9 (1.9)	0.002
**Age (years)**			
19–30	22.8 (0.8)	19.4 (2.2)	0.079
31–50	32.8 (0.8)	30.6 (2.2)	0.306
>50	44.4 (1.1)	49.9 (2.8)	0.032
**Ethnicity**			
Non-Hispanic whites	47.8 (3.1)	44.2 (4.2)	0.135
Non-Hispanic blacks	21.4 (2.2)	31.8 (4.2)	0.001
Mexican Americans	19.3 (1.6)	11.7 (2.2)	<0.001
Hispanics	7.4 (1.2)	8.1 (1.9)	0.548
Others	4.0 (0.4)	4.1 (1.1)	0.914
**Education**			
<High school	28.3 (1.0)	23.9 (1.9)	0.022
High school/GED	23.5 (0.6)	23.9 (1.9)	0.858
Some college	18.5 (1.0)	19.1 (2.1)	0.729
≥College graduate	25.8 (0.6)	29.4 (2.2)	0.125
Missing education	3.9 (0.3)	3.6 (0.9)	0.734
**Total physical activity**			
Quartile 1 [lowest]	24.7 (0.7)	24.4 (1.7)	0.877
Quartile 2	20.9 (0.5)	22.4 (1.9)	0.426
**Total physical activity**			
Quartile 3	18.8 (0.5)	20.0 (1.3)	0.406
Quartile 4 [highest]	17.9 (0.6)	18.2 (1.5)	0.793
Missing PA	17.7 (0.6)	14.9 (1.9)	0.130
**Cranberry juice intake**			
mL/day	0 (0)	221.1 (8.1)	<0.001
oz/day	0 (0)	7.5 (0.3)	<0.001

* Cranberry beverage consumers were identified as anyone who reported consuming cranberry juice products over two non-consecutive 24-h dietary recalls; ^†^ *P*-value derived from chi-squared tests comparing cranberry juice consumers to non-consumers.

Important differences in macronutrient intakes were also noted (Table 2). Adjusted for gender, age, and race, CB consumers compared to non-consumers had higher total daily energy intake and intake of total sugar (+12 g/day) and higher intake of carbohydrates, both in absolute (+15.6 g/day) and relative (as a percent of daily energy) terms (Table 2). There was no significant difference in weight or BMI between CB consumers and non-consumers, however, and CB consumers were more likely to be normal weight than CB non-consumers (33.1% *vs.* 30.0%, respectively; *p* < 0.001) and they had significantly lower waist circumferences (*p* = 0.001) (Table 2).

**Table 2:** Fully adjusted differences in macronutrient intake * and anthropometric outcomes between adult cranberry beverage consumers ^†^ and non-consumers, NHANES 2005–2008.

Outcome	Cranberry Beverage		*P*-Value ^‡^
	Non-Consumers	Consumers	
**CB consumption, mean (SE)**			
mL/day	0	221.1 (0.2)	<0.001
fl oz/day	0	7.5 (0.01)	<0.001
**Macronutrients, mean (SE)**			
Total Energy (kcal/day)	2046 (16)	2086 (42)	0.309
Carbohydrates (g/day)	251.2 (1.8)	266.8 (4.7)	0.002
Protein (g/day)	79.9 (0.5)	79.8 (1.5)	0.979
Total Fat (g/day)	76.6 (0.7)	75.7 (1.7)	0.545
Total Sugar (g/day)	114.6 (0.9)	126.5 (2.2)	<0.001
**Percent daily energy, % (SE)**			
Carbohydrates	49.7 (0.06)	51.9 (0.09)	<0.001
Protein	16.0 (0.02)	15.6 (0.03)	<0.001
Total Fat	33.2 (0.06)	32.0 (0.09)	<0.001
Sugars	22.5 (0.03)	24.4 (0.08)	<0.001
**Anthropometrics, mean (SE)**			
Weight (kg)	81.1 (0.16)	80.1 (0.35)	0.001
BMI (kg/m^2^)	28.8 (0.04)	28.5 (0.8)	0.001
Waist Circumference (cm)	98.4 (0.10)	97.4 (0.27)	0.001
**BMI Distribution, % (SE)**			
Normal weight	30.3 (0.24)	33.1 (0.42)	<0.001
Overweight/Obese	54.2 (0.34)	50.7 (0.60)	<0.001

* Estimates are predicted from multivariable linear (macronutrient and anthropometrics) and logistic (BMI distribution) regression models adjusting for age, gender, race/ethnicity, highest level of education completed, total physical activity, and total energy intake (not included for models with macronutrients expressed as a percent of total energy). All results are weighted to be nationally representative; ^†^ Cranberry beverage consumers were identified as anyone who reported consuming any cranberry juice product at least once over two non-consecutive 24-h dietary recalls; ^‡^ *P*-value derived from student’s *t*-test or chi-squared tests (BMI distribution only) of fully adjusted (predicted) means comparing cranberry beverage consumers to non-consumers.

Macronutrient intakes were significantly different across levels of CB consumption (Table 3). There was a statistically significant increasing trend of total energy (*p* for trend < 0.001), and percent of energy from carbohydrates (*p* for trend < 0.001), and total sugar intake (*p* for trend < 0.001) moving from non-consumers up through the highest level of intake, while there was a decreasing trend in percent energy from total fat and protein (*p* < 0.001, Table 3). However, significant differences in anthropometric measures were not observed across increasing level CB consumption (*p* for trend >0.05, Table 3).

**Table 3:** Adjusted means for macronutrient and body composition measures across levels of cranberry beverage consumption *, NHANES 2005–2008.

Outcome	Non-Consumers	Consumers			*p*-for Trend
		Low	Middle	High	
**Sample size**	9753	385	152	44	-
**CB Consumption**					
Range					
mL/day	0	<240 mL	240–469 mL	≥470 mL	-
oz/day	0	<8 oz	8–15.9 oz	≥16 oz	-
Mean (SE)					
mL/day	0	126.1 (0.06) ^†^	313.0 (0.12) ^†^	734.4 (0.18) ^†^	<0.001
oz/day	0	4.3 (0.002) ^†^	10.6 (0.004) ^†^	24.8 (0.006) ^†^	<0.001
**Macronutrients, mean (SE)**					
Total Energy (kcal/day)	2046 (16)	1985 (43)	2251 (77) ^‡^	2403 (107) ^‡^	0.672
**% Energy from**					
Carbohydrates (g/day)	49.7 (0.06)	51.1 (0.09) ^†^	53.3 (0.15) ^†^	54.1 (0.31) ^†^	<0.001
Protein(g/day)	16.0 (0.02)	16.0 (0.03)	15.0 (0.05) ^†^	14.3 (0.07) ^†^	0.001
Total Fat (g/day)	33.2 (0.06)	32.6 (0.11) ^†^	31.3 (0.12) ^†^	30.8 (0.19) ^†^	<0.001
Total Sugar (g/day)	22.5 (0.03)	23.4 (0.09) ^†^	26.0 (0.12) ^†^	28.4 (0.21) ^†^	<0.001
**Anthropometrics, mean (SE)**					
Weight (kg)	81.1 (0.2)	79.2 (0.3) ^†^	81.2 (0.6)	83.1 (1.3)	0.420
BMI (kg/m^2^)	28.8 (0.04)	28.5 (0.1) ^†^	28.5 (0.2) ^§^	29.2 (0.2)	0.273
Waist Circumference (cm)	98.4 (0.1)	97.2 (0.3) ^†^	97.2 (0.4) ^‡^	97.9 (0.8)	0222
**BMI Distribution, % (SE)**					
Normal weight	30.4 (0.2)	32.3 (0.4) ^†^	36.5 (0.9) ^†^	29.5 (1.4)	0.162
Overweight/Obese	53.9 (0.4)	50.0 (0.8) ^†^	49.5 (1.2) ^†^	53.6 (2.7)	0.260

* Estimates are predicted from multivariable linear (continuous outcomes) and logistic (categorical outcomes) regression models adjusting for age, gender, race/ethnicity, highest level of education completed, total physical activity, and total energy (total energy is not included in models with nutrient outcomes expressed as a percent of total daily energy). All results are weighted to be nationally representative. Cranberry beverage consumers were identified as anyone who reported consuming any cranberry juice product at least once over two non-consecutive 24-h dietary recalls; ^†^ Estimate is statistically significantly different from non-consumers using student’s *t*-test, *p* < 0.001; ^‡^ Estimate is statistically significantly different from non-consumers using student’s *t*-test, *p* < 0.01; ^§^ Estimate is statistically significantly different from non-consumers using student’s *t*-test, *p* < 0.05.

For all macronutrient and anthropometric outcomes, statistically significant differences were observed comparing low, middle, and high CB consumption to non-consumers (Table 3). The highest consumers reported 350 more calories per day compared to CB non-consumers (*p* < 0.001) and roughly 2% less energy from fat (*p* < 0.01, Table 3). Daily intake of carbohydrates and total sugars were greater at the top two levels (middle and high) of CB consumption compared to CB non-consumers, a difference of roughly 4% and 6% between the highest consumers and non-consumers, respectively (Table 3).

At the lowest level of CB consumption, weight was lower compared to non-consumers (79.2 *vs.* 81.1 kg, respectively, *p* < 0.001, Table 3). Similarly for BMI the lowest and middle level had significantly lower values compared to non-consumers (*p* < 0.01). Despite consumption of greater calories and total sugars (Table 2), there was no significant difference in any of the anthropometric outcomes among persons at the highest level of CB consumption compared to non-consumers.

## 4. Discussion

Numerous clinical studies evaluating the association between cranberry juice consumption with urinary health [20,21,22,23,24,25,26], antioxidant status [27,28], or cardiovascular disease [31,32,33,39], document the health benefits of consuming flavonoids in the form of cranberry products. To our knowledge however, studies evaluating trends in consumption of cranberry beverages, or their association with other markers of health (e.g., anthropometry, dietary status) do not exist. Our results demonstrate that although cranberry juice consumers had slightly higher average daily total energy intakes (40 kcal) and higher macronutrient intakes for carbohydrates and total sugars compared to non-consumers they did not have higher weights or level of BMI and actually had a higher likelihood of being normal weight compared to non-consumers. Furthermore, at the highest level of intake, the probability of being overweight/obese was no different than for non-consumers.

Although historically there has been a period of increased consumption of added sugars [4,40], with SSBs among the top contributors [4,6,7], recent data suggest that this trend may be reversing. US adults report a decline in total energy intake between 2003 and 2010 [41] and energy from SSBs has decreased significantly for both adolescents and adults between 1999 and 2010 [42]. These observational findings mirror data which show declines in the availability of total energy, carbohydrates, and sugars [43].

In the present study, CB consumers intake averaged roughly 7.5 oz/day which is consistent with most recommendations for servings of juice and provides a daily flavonoid intake of 125–150 mg/day based on 27% CB juice [44]. Current estimates of flavonoid intake in the US population ranges from 190–210 mg/day [45,46]. Thus, the simple dietary behavior of consuming a single serving of a cranberry beverage daily would almost double the US population intake of flavonoids, an important group of compounds that have been linked to numerous health benefits [20,21,22,27,31,32,33].

Despite the ongoing controversy around SSBs and health, observational and epidemiological data support 100% fruit juice consumption as part of an overall healthy lifestyle. Importantly, 100% juices contain micronutrients and vitamins which are vital to supporting and maintaining health. In a cross-sectional study among US adults, for example, Pereira *et al.* found that compared with non-consumers, those who consumed 100% fruit juice were leaner and had lower odds of obesity [47] and consumers of 100% orange juice, in particular, have been shown to have lower levels of BMI, waist circumference, and percentage body fat, and lower odds for overweight and obesity compared to non-consumers [16]. The results from the present study are similar to those reported for other 100% fruit juices, with cranberry juice consumers having lower waist circumferences and lower risk of obesity compared to non-consumers.

The present study is not without limitations. First, our results are based on cross-sectional data and thus causality cannot be determined. Second, although we examine associations with, and account for, total energy intake, we do not have measures of an individual’s entire diet and thus cannot account for possible influences of other components of dietary intake on our outcomes of interest. It remains a possibility that CB consumption is simply a marker for other healthy dietary behaviors and that these behaviors, rather than the CB consumption in and of itself, are at least partially responsible for our observed associations with anthropometric outcomes. In sensitivity analyses we further included control for a select number of food groups and our predicted outcomes were unchanged. Third, dietary intake was assessed using the average of just two 24-h dietary recalls, which although it is not necessarily representative of usual intake at the level of the individual, is widely accepted as a method for capturing and estimating average consumption across a population. Lastly, as with all observational studies, it is possible that there are unmeasured factors which could account for these observed associations. There is no way to anticipate how these unmeasured factors may affect our observed associations. There are several strengths as well. The diet data are collected using the automated multiple pass method which improves recall and all results are nationally representative which allows us to draw conclusions about the larger US adult population.

## 5. Conclusions

Cranberries contain significant amounts of flavonoids and polyphenolic compounds which are positively associated with health, and cranberry juice beverages are rich in these bioactive compounds. However, because of their inherent tartness cranberries are considered unpalatable in their raw state and thus juice processing requires the addition of nutritive and non-nutritive sweeteners to enhance palatability. Because of this, they are frequently perceived as SSBs. However, cranberries beverages have similar or lower calories and similar or higher levels of polyphenols than 100% juices [44,48]. In addition, results from the present study suggest that there may be a role for consumption of cranberry juice beverages as part of a healthy diet and lifestyle, despite the fact that they are sweetened with nutritive sweeteners, especially in light of the growing body of evidence showing that consumption of polyphenol-rich cranberry juice is linked to improved measures of cardiovascular health [31,49,50].
